# Epigenetics in Tuberculosis: Immunomodulation of Host Immune Response

**DOI:** 10.3390/vaccines10101740

**Published:** 2022-10-18

**Authors:** Avinash Khadela, Vivek P. Chavda, Humzah Postwala, Yesha Shah, Priya Mistry, Vasso Apostolopoulos

**Affiliations:** 1 Department of Pharmacology, L. M. College of Pharmacy, Ahmedabad 380009, India; 2 Department of Pharmaceutics and Pharmaceutical Technology, L. M. College of Pharmacy, Ahmedabad 380009, India; 3 PharmD Section, L. M. College of Pharmacy, Ahmedabad 380009, India; 4 Immunology and Translational Research Group, Institute for Health and Sport, Victoria University, Melbourne, VIC 3030, Australia; 5 Immunology Program, Australian Institute for Musculoskeletal Science (AIMSS), Melbourne, VIC 3021, Australia

**Keywords:** *Mycobacterium tuberculosis*, epigenetics, histone modification, DNA methylation, miRNAs

## Abstract

Tuberculosis is a stern, difficult to treat chronic infection caused by acid-fast bacilli that tend to take a long time to be eradicated from the host’s environment. It requires the action of both innate and adaptive immune systems by the host. There are various pattern recognition receptors present on immune cells, which recognize foreign pathogens or its product and trigger the immune response. The epigenetic modification plays a crucial role in triggering the susceptibility of the host towards the pathogen and activating the host’s immune system against the invading pathogen. It alters the gene expression modifying the genetic material of the host’s cell. Epigenetic modification such as histone acetylation, alteration in non-coding RNA, DNA methylation and alteration in miRNA has been studied for their influence on the pathophysiology of tuberculosis to control the spread of infection. Despite several studies being conducted, many gaps still exist. Herein, we discuss the immunopathophysiological mechanism of tuberculosis, the essentials of epigenetics and the recent encroachment of epigenetics in the field of tuberculosis and its influence on the outcome and pathophysiology of the infection.

## 1. Introduction

Mycobacterium tuberculosis (Mtb) is a major worldwide concern for public health, being mainly a poverty-based disease that affects the most vulnerable in the population [1]. Approximately one third of the world’s population are or have been infected by Mtb. Globally, infectious diseases still account for 25% of deaths, only surpassed by cardiovascular diseases [2]. The World Health Organization (WHO) reported that an estimated 8.6 million people contract Mtb annually, of which two thirds are males. The mortality of 1.3 million patients sees 320,000 cases related to concurrent HIV infection [3]. The current average annual incidence of smear positive Mtb stands at 84 per 100,000 cases annually in India [4]. There has been a decrease in the incidence of Mtb infections in the United States of America since 2010 by almost 2–3% [5]. Apart from this, Africa accounts for a quarter of newly diagnosed Mtb cases globally and for more than 25% of Mtb infection-related deaths.

The main goal of pharmacological management of Mtb includes prevention of disease transmission and sterilization of the lesion [6]. The current treatment protocols for first line regimen remains with isoniazid, rifampicin, pyrazinamide and ethambutol, which are either used on their own or in combination [7]. As expected, Mtb bacilli have undergone several mutations, which are generally infrequent, although this leads to poorer responses when treated with single agents [8]. Thus, when a combination of drugs are used, it resolves the problem of resistance as well as enhances the efficacy of the drugs as they work via different mechanisms. For multidrug resistant infections, however, which do not respond to first line agents, a group of drugs defined as the second/third line agents are used which are more toxic, expensive and less efficacious when compared to first line agents [9]. Newer agents for the treatment of multi drug resistant Mtb infections include bedaquiline and delamanid [10]. Another approach to prevent the widespread infection of Mtb is the development of a vaccine derived from an attenuated strain of *M. bovis*, which has been in use since the 1930s [11]. The bacillus of Calmette and Guerin (BCG) vaccine acts by providing accelerated immune responses and thus reducing the spread of infection [12]. There are a number of issues that act as obstacles for the management of Mtb infections, for example, social stigma, marginalization, poor adherence to treatment due to prolonged duration [13,14]. In addition, immune impairment, for example, those with HIV infection, further increases risk of infection and concurrent infections.

Several newer strategies are currently under development to overcome the problems with the current standard of care. Interferon (IFN)α [15], IFNγ [16], imiquimod [17], interleukin (IL)-12 [18], granulocyte-monocyte colony stimulating factor (GM-CSF) [19], levamisole [20] are some agents that are under consideration as anti-Mtb treatments. Along with this, a vaccine derived from *M. vaccae*, which is a rapidly growing saprophytic mycobacterium, is also being evaluated as a novel approach against Mtb infections but has shown variable results thus far [21]. In an attempt to prevent the problem of treatment adherence, which has been one of the major reasons of relapse and development of resistance, the WHO has recommended a method of supervision known as ‘directly observed treatment short course’ since the 1990s, which has shown improved outcomes in terms of adherence to treatment and efficacy [22,23,24].

In recent times, personalised therapy (also called host directed therapy) for conditions such as Mtb are being developed. In order to develop the personalised host directed therapy, the understanding of epigenetic processes can play a distinguishing role [25]. Some recent studies have reported epigenetic changes such as histone modification, DNA methylation and miRNA mediated regulation brought through Mtb pathogen interaction with the host’s cells. The role of various cytokines such as IFN-γ, IL-12 and TNF-α to induce acetylation and methylation on histone proteins can be explored to develop novel therapeutic strategies. The field of targeting epigenetic modifications known as epi-therapeutics is new and requires lot of work in order to develop successful therapy [26]. Herein, we present the immunopathophysiology of Mtb infection and the epigenetic modification brought by Mtb pathogen and recent in vitro and in vivo experiments, which can be explored further to develop successful therapies using epigenetics.

## 2. Immuno-Pathophysiology of *Mycobacterium tuberculosis*

Mycobacterium tuberculosis pathogen is predominantly present in the environment in the form of aerosol and it gains access into the body via the lungs through inhalation [27]. Its presentation to the host body depends on the virulence of the pathogen and the host’s immune system [26]. This can be explained by its immune-pathophysiological mechanism including both arms of the immune system, the innate and adaptive immune response. If the Mtb pathogen manages to invade the physical barrier, then it is presented to the innate immune system of the host. As such, invasion of Mtb leads to substantial changes in the host immune cells and activates the innate immune response as the first line of defense [28]. Various immune cells are involved as the response to infection; however, in the case of Mtb infection, the first cells to encounter the pathogen are macrophages [29]. Pathogens that enter the body express pathogen-associated molecular patterns, which have conserved motifs that serve as ligands for host pattern recognition receptors (PRR) including toll-like receptors (TLRs) and, in turn, gives rise to danger-associated molecular patterns. As such, these molecular patterns aid the immune system to elicit an innate immune response against the invading Mtb pathogen [30]. In addition, the pathogen has several ligands on its surface including mannose, lectin, surface protein A, cluster of differentiation (CD)14, which are recognized by PRRs [25]. The PRRs can be cataloged mainly into TLRs, c-type lectin receptors, nod-like receptors, retinoic acid inducible (RIG)-I like receptors, complement receptors (1, 3, 4), mannose receptor, scavenger receptor, CD14 and CD43 [31]. Of all the PRRs, TLR-1, -2, -4, -9, play an important role in the pathogenesis of the Mtb. TLR-1, -2, -4 are presented on both immune cells and non-immune cells whereas TLR-9 is presented only on immune cells. Furthermore, the inflammasome pathway is instigated by PRRs, which play an important role in innate immune mechanisms via the regulation of caspase-1 in response to pathogen associated molecular patterns and danger associated molecular patterns recognition by host cells [32]. Mtb also aggressively stirs up the assembly of pentraxin-3 (PTX-3), a 42 KDa soluble PRR involved in acute immune responses towards infection [33]. In adaptive immune responses, B and T cells (CD4+, CD8+, regulatory T (Treg)), NK cells, macrophages and dendritic cells are involved in Mtb infections. The recruitment of NK-cells, granulocytes and activated macrophages (via lysosomes and ROS) is important for killing the pathogen, and leads to the hallmark characteristic of Mtb formation of the granuloma [34,35]. In addition, Mtb primed immune cells secrete pro-inflammatory cytokines, IL-1, IL-6, IL-12, IL-18, IFNγ, and TNFα.

The excessive stimulation by IFNγ over time leads to the conversion of activated macrophages to epithelioid macrophages (long elongated nucleated cells). They surround the infection in the lungs and form the granuloma. Within the granuloma there are high levels of TNFα, which limit the infection [36], as well as central caseating necrosis, which is surrounded by epithelial macrophages, lymphocytes and fibroblasts (produce collagen around caseating necrosis). Further, the level of 1-alpha hydroxylase is upregulated at the site of granuloma, which leads to its calcification [35,37]. If Mtb remains within the granuloma, the infection is termed latent Mtb infection. When host immunity is compromised, it leads to the reactivation of Mtb, known as secondary tuberculosis [38]. This is mediated by type-4 delayed hypersensitivity reaction. Moreover, Tregs are present that suppress the mycobactericidal activity of immune cells with the help of IL-10, IL-35, TGFβ. Thus, the interaction between the Mtb pathogen and the host’s immune cells contributes to the immune-pathogenesis of Mtb [26,39]. In addition, Mtb bacillus specifically exploits the moonlighting functions of PE proteins by utilizing immune signalling pathway, which subsequently helps in growth as proliferation of the pathogen inside the host. These PE6 proteins, by binding to the iron inside the cell, help progression of the disease via pathogenic proliferation and intracellular pathogenic survival. Vitamin D also plays a role in the pathogenesis of Mtb infections, as it leads to the formation of cathelicidins and defensins, which contribute to the destruction of the pathogen [40].

## 3. Tuberculosis and Epigenetic Regulations and Modifications

The epigenetic concepts were developed in the 1940s by CH Waddington, as a process influencing genetic outcomes without changing cell sequencing [41]. Epigenetic regulations include any alteration in gene expression due to chromosomal modifications without actually modifying the sequencing of nucleotides in the coding DNA [42]. The Mtb pathogen takes over and reprograms the host epigenome via histone modifications, DNA methylation and miRNA mediated regulation of genes as a self-protective mechanism [43,44]. These modifications play an important role in Mtb-induced host immunomodulation [26]. The tubercular bacilli induces various perturbations in the epigenetic regulations, specifically in those mediated by DNA methylation [45]. Using multiple cohorts, tissue types and transcriptomic analysis showed that epigenetic changes associated with Mtb induces oxidative stress. This leads to premature cellular ageing and induces senescence [45]. These induced changes by the bacteria are reversible and, as such, can be easily an effective target for therapeutics. IFNγ induces expression of HLA-DR α/β mRNA, which is inhibited by the Mtb-bacilli along with partial inhibition of CIITA expression in the Mtb infected macrophages without having any effect on the expression of IFN regulatory factor-1 mRNA [46]. Epigenetic mechanisms regulate the transcriptional profile of immune system related genes contributing to the interaction between the host and the infectious agent [47]. More recently, it was reported that the sensitive Mtb strains lead to sufficient immune activation in the host to clear the infection but the resistant Mtb strains cause sub-optimal immune induction by enabling better intracellular survival of bacilli by over-expressing genes, inducing host lipid metabolism [48].

The immune cells which are predominantly involved in these modifications induced by Mtb pathogen include T cells and macrophages. Both T effector and T helper cells play important roles in the epigenetic pathology of Mtb. CD4 promotor is responsible for epigenetic repression of matured CD8+ T cells. In addition, Tregs are capable of inducing histone modifications as well as DNA methylation on various transcription factors including FOXP3. MHC II expression and subsequent CIITA role are highly influenced by epigenetic changes such as DNA methylation. Macrophages also play an important role in the pathogenesis of Mtb. These are sensitive to histone methylation-based epigenetic changes in the host cells.

### 3.1. Histone Modifications

Histone proteins widely known as chromatin remodeling proteins that are responsible for formation of the nucleosome octamer around the DNA after around 1.7 turns. These histone proteins are responsible for normal structural integrity of chromatin. Eight different classes of post-translational histone modifications with 60 sites for modifications are present [49]. These include proline isomerization, arginine deamination, lysine/arginine methylation, serine, glutamate poly-ADP ribosylation, threonine/tyrosine, ubiquitination, sumolylation and lysine acetylation [50]. All these modifications are covalent in nature and are guided by various enzymes such as kinases, phosphatase, histone deacetylase (HDAC), histone acetyl transferase (HAT), histone methyltransferases (HMTs) and histone demethylase (HDMs) [51].

#### 3.1.1. Histone Methylation

Methylation occurs on lysine or arginine residues of the histones, and leads to activation or suppression of the genes, respectively. Methylation can take place in mono, di- or trimeric forms each having different consequences. Around 90% of methylation takes place at the cytosines of CpG sites in human somatic cells [52]. Methylation at CpG, due to which gene expression is silenced as a result of prevention of association of transcription or DNA binding factors to their respective binding sites by recruiting co-repressors in order to methylate CpG nucleotides, leads to chromatin modification into its repressor forms [53]. Rv1988 is a mycobacterial histone methyltransferase secretary protein which demethylates the amino acid arginine specifically at 42nd position in the histone H3, which has a profound effect on host gene transcription due to its capacity to localize with the chromatin in the host nucleus. This Rv1988 is known to repress gene expression by methylating histone H3 at R42 [54]. A similar finding has also been available for other mycobacterial proteins such as Rv2966c. This type of proteins loosens the host defense by decreasing the effect of genes with protective activity, thus acting as first line of attack during infection [54]. Another report showed the induction of H4K20me1 and its regulation of apoptosis and subsequent inflammation in assisting Mtb survival induced by histone methyl transferase SET8 [55]. The result of these methylation modifications depending on their nature, suppress gene expression, alter host histones via controlling of the cell signaling, etc. For instance, the downregulation of KDM6B gene induced by Mtb pathogen leads to hypermethylation of the host histone protein H3K27.

#### 3.1.2. Histone Acetylation

Addition of an acetyl group to the lysine site leads to modifications in DNA expression. Any molecule that can mediate the addition of this acetyl group on the host proteins are termed as HATs. In this context, the Mtb antigenic proteins acts as HATs and induces acetylation changes not just on histones but also on non-histone proteins. NF-κB p65 is an example of non-histone acetylation modification. The inflammatory response against the Mtb is mediated by various host enzymes such as matrix metalloproteinase (MMP), a zinc-dependent endopeptidase that plays a crucial role in the development of cavitation. On acetylation of these MMP proteins, there is intracellular survival of Mtb bacilli in the host tissue. They are secreted by Mtb-infected monocytes and macrophages along with non-infected stromal cells. The epigenetic mechanisms act as important regulators of MMP activity in various non-infectious as well as infectious diseases including Mtb [56,57]. The implications of epigenetics in induction of MMP- 1/3, especially by histone acetylation associated with transcriptional activation, is investigated and demonstrated in a study. The class 1 HDAC expression is also suppressed by Mtb infection in macrophages [58]. HDACs are known to be negative regulators for the expression of genes [59]. Thus, MMP upregulation in Mtb infection leads to lung tissue damage and the HDAC and HATs activity is required for induction in expression of MMPs. This MMP-induced tissue breakdown is the most important contributor in immune pathophysiology as well as host epigenetic changes [58]. The observed changes in gene transcription during development are due to histone modifications such as methylation in the arginine residues of histone H3 [60].

**Figure 1:**
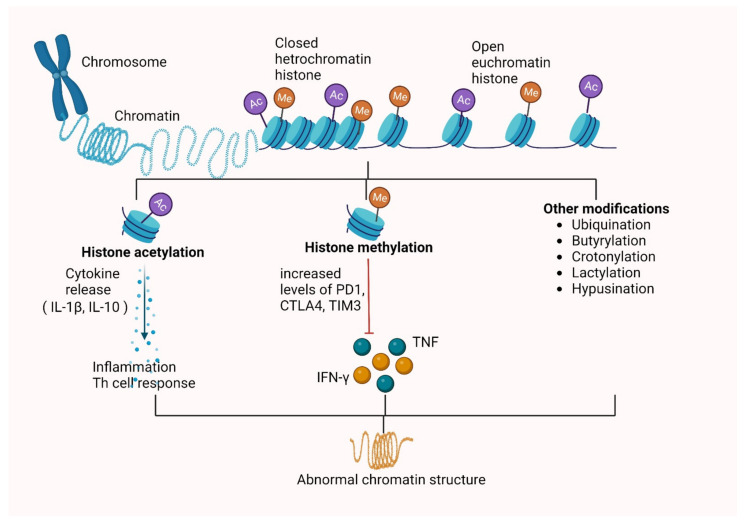
Consequences of *Mycobacterium tuberculosis* induced methylation, acetylation and other histone modifications in the host’s immunity. Abbreviations: Ac: acetylation, Me: methylation, IL-1β: interleukin 1-beta, PD1: programmed cell death-1, CTLA4: cytotoxic T-lymphocyte–associated antigen 4, IFN-γ: interferon gamma, TIM3: T-cell immunoglobulin and mucin domain 3, TNF: tumor necrosis factor.

Several HDACs, such as HDAC1, HDAC2, HDAC3 and sirtuins, catalyze histone deacetylation out of which HDAC3 predominantly causes deacetylation during Mtb infection [61]. The suppression of IL12B expression by Mtb via HDAC1 is also shown [62]. Host gene expression is suppressed by HDAC-induced deacetylation, whereas HAT-induced histone tail acetylation leads to chromatin activation by increasing spacings between the nucleosomes [63]. Other proteins, such as ESAT-6 and LpqH, inhibit MHC-II expression and antigen presentation by inducing histone modifications [64]. Mtb infections also inhibit various IFN-induced genes such as HLA-DR, CD64, CIITA trans-activator irrespective of normal JAK-STAT1 signaling cascade activation [65]. Similarly, another mycobacterial protein Rv3763 is responsible for histone hypoacetylation at CIITA promoter via suppressing INF-γ induced genes. The interaction of Rv2966c with the macrophage epigenome affects the non-CpG methylation at specific loci and is also known [66]. Thus, epigenetic modifications on histones requires proper homeostasis as it has a pivotal role for proper gene expression regulation. The mechanism of the consequences of Mtb-induced histone modifications are summarized in Figure 1.

### 3.2. Alteration in Expression of Non-Coding RNAs

**Figure 2:**
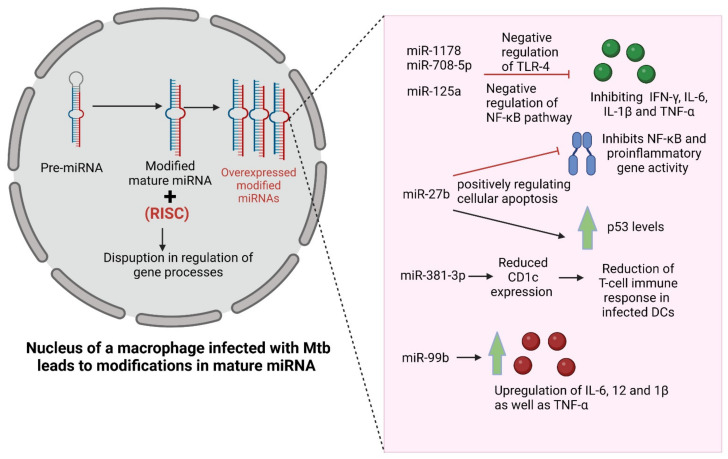
Mechanism of induction of changes due to Mtb infection-induced modifications in the miRNA. Abbreviations: miRNA: micro-RNAs, RISC: RNA-induced silencing complex, Mtb: mycobacteriaum TB, TLR: toll-like receptors, NF-ΚB: nuclear factor kappa B, IFN-γ: interferon gamma, IL: interleukin, CD1c: cluster of differentiation, DC: dendritic cells, TNF: tumor necrosis factor.

The proteins produced as a result of translational processes are dependent on the code sequences present on the RNA templates. However, after the sequencing of the entire mammalian genome and transcriptome was published, it was noted that some of the sequences of the RNA were not reflected in the coded proteins [67]. Thus, these were categorized as non-coding RNAs (nc-RNAs), also known as micro RNAs (miRNAs) [68]. miRNAs basically play a role in regulation of bacterial infections; thus, its dysregulation leads to various infectious diseases as well as immune disorders [69]. These nc-RNAs, approximately 22 nucleotides in length, have a considerable regulatory role in certain cellular mechanisms such as DNA methylation as well as histone modifications and other, around one-third of mammalian genes [70]. These miRNAs are basically endogenously acting gene silencers that repress gene expression at a translational level by targeting mRNA [71]. Important cellular pathways such as cell proliferation, angiogenesis, invasion is regulated by these miRNAs along with other complex epigenetic mechanisms such as dynamics of chromatin structures and genome organization in nucleus [72]. All these regulation of gene processes are a result of a combination of mature miRNAs and RNA-induced silencing complex [73]. miRNAs are also involved in regulation of the immune response against Mtb of dendritic cells (DCs). The role of miRNAs in patients with Mtb infections and TB patients was proved by a study showing overexpression of more than 59 miRNAs in TB patients’ serum compared to healthy controls [74]. Moreover, after being infected by Mtb, host macrophages induce a particular modulation of miRNA [75]. During Mtb infection, miRNA mediates various signaling pathways as well as apoptosis and autophagy. For instance, miR-1178 and miR-708-5p when overexpressed, it negatively regulates TLR-4, resulting in inhibition of expression of proinflammatory mediators such as IFN-γ, IL-6, IL-1β and TNF-α [76,77]. miR-125a upregulation leads to negative regulation of NF-κB pathway via TRAF6, ultimately leading to blockage of proinflammatory cytokines [78]. miR-27b is triggered by the Mtb-induced TLR2/MyD88/NF-κB pathways, which further inhibits NF-κB and proinflammatory gene activity while increasing p53, thus positively regulating cellular apoptosis [79]. miR-381-3p mediates reduced CD1c expression and subsequent reduction of T-cell immune response in Mtb infected DCs [80]. Moreover, miR-99b is highly expressed in Mtb-infected DCs, which upregulate the expression of inflammatory mediators such as IL-6, 12 and 1β, as well as TNF-α, thus activating DCs to clear the phagocytic Mtb [81]. Other than these, miR-let-7f, miR-132, miR-26a, miR-20b, miR-155, miR-21, miR-146a are also important in regulation of cellular pathways, as briefly summarized in Figure 2. There are many host ncRNAs that are not yet discovered with unknown functions and regulatory networks due to their complexity, which still needs to be studied further [82].

### 3.3. Alterations in DNA Methylation

DNA methylation takes place when Mtb induces the transfer of a methyl group from cytosine base (carbon 5 position) mediated by DNA methyltransferase enzymes to form 5-methylcytosine [83]. It also plays an important role in different cellular processes such as differentiation, development, reprogramming, as well as gene silencing that leads to induction of diseases including TB [84]. It was shown in a study that the methylation Mtb immunity-related genes are upregulated, which leads to suppression of immune response against Mtb, which indicated DNA methylation to be an important epigenetic target [85]. It was demonstrated that MamA, an adenine methyltransferase, is encoded by Rv3263 and responsible for all detectable methylation modification by a particular Mtb strain, H37Rv. The effects of these mycobacterial Rv proteins are summarized in Table 1

**Figure 3:**
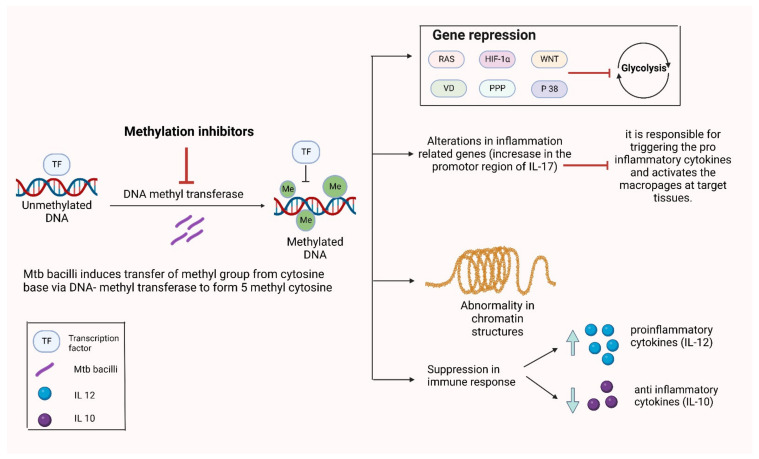
Induction of DNA-methylation by Mtb and its subsequent effect on the host immunity. Abbreviations: TF: Transcription factor, DNA: Deoxy ribonucleic acid, Me: Methylation, IL: Interleukin, RAS: Rat sarcoma virus, HIF: Hypoxia inducible factor, WNT: Wingless-related integration site, VD: Vitamin D, PPP: Pentose phosphate pathway.

The contribution of methylation-mediated regulatory pathways in showing lineage specific characteristics seen in different strains of Mtb is proven [87]. Thus, the interference of Mtb with host epigenome as an aid to survival is very well known. A study report identifies over differentially methylated regions (DMRs) in Mtb infected macrophages. This proves the use of non-canonical strategies by Mtb bacilli to establish infection [88]. It was indicated in a study by genetic ontology analysis; the contribution to many immune-biological functions such as immune cell activation and regulation as well as cellular response to IFN-γ and cytotoxicity by DMR-associated genes [89]. This epigenetic modification is quite stable compared to the histone modification’ thus, it is quite less reversible process leading to silencing of the gene expression for a much longer time. DNA methylation shows dynamic effects on the Mtb infected DCs. Significant changes in the DMRs majorly at low CpG density regions as well as in distal regulatory or enhancer regions after Mtb infection in DCs compared to uninfected cells [90]. Around 40% of miRNAs were differently expressed in Mtb-infected DCs [91]. Mtb-infected macrophages lead to significant methylation level alterations in inflammatory related genes showing quite a larger increase in the promoter region of IL-17 compared to other receptors in infected macrophages, as shown in Figure 3. Moreover, these methylation pattern changes depend on the type of Mtb strain as well as host genotype [92]. Early secreted antigen 6 (ESAT6) has a significant role in reducing IFN-γ induced methylation of histone H3K4 and acetylation of CIITA pl locus histone. Furthermore, inhibition of type IV CIITA expression was found to be TLR-2-dependent; on the other hand, type I CIITA expression inhibition was TLR-2-independent, both mediated by ESAT6 [93]. Methylation was increased in Mtb-infected monocytes, which presented a reduced anti-inflammatory cytokine IL-10 secretion and an increased proinflammatory cytokine IL-12 secretion, which indicates impaired monocyte ability in regulating excess inflammation, which leads to lung injury [94]. Enhanced intracellular survival (Eis) protein secreted by Mtb leads to increased survival of infected macrophages by acetylation of histone proteins [95]. Thus, the susceptibility of the Mtb bacilli depends on the methylation status that varies in different ethnic groups [96].

## 4. Therapies Targeting Epigenetic Modifications for *Mycobacterium tuberculosis*

Targeting epigenetics works on the concept of targeting the host instead of the Mtb bacilli directly, also known as HDT (Table 2) [97].

It has been reported that broad chemical inhibition of HDAC enhances anti-microbial response against Mtb by differentiating macrophages into a comparatively stronger bactericidal phenotype along with decreased secretion of inflammatory cytokines. A highly accepted in vivo model for inhibition of HDAC in M-marium-infected zebrafish embryos significantly reduces the mycobacterial burden, whereas selective class IIa inhibition leads to a prominent decrease in bacterial outgrowth [98]. HDAC inhibitors such as suberoylandilide hydroxamic acid (SAHA), marketed as vorinostat and trichostatin A (TSA), are looked forward to as they provide quite improved bacterial clearance by modulating the epigenetic modifications induced by Mtb. These show mixed effects as, along with resolving inflammation, they also impair microbicidal activities of macrophages by impairing its functions [99]. This is achieved by reducing the levels of reactive oxygen species (ROS) as well as nitric oxide (NO) in the exposed macrophages [100]. Another nonspecific HDAC inhibitor phenylbutyrate when used in combination with vitamin D acts as a dual targeted therapy against the host as well as the pathogen [97]. SAHA is also known to increase IL-1β levels and decrease IL-10 levels in host alveolar macrophages and monocyte-derived macrophages along with enhanced exhibition of IFN-γ and GM-CSF coproduction [101]. These older broad spectrum HDAC inhibitors have a quite wider site of action and targets several different HDACs. By contrast, another specific HDAC-6 inhibitor tubustatin was studied in mice and has been associated with improved resolution of bacteraemia, less organ dysfunction as well as modulated stress response by increasing monocyte and neutrophil counts and reversing lymphopenia [102,103]. It has more specific actions and has greater selectivity. As opposed to broad spectrum HDAC inhibitor, tubustatin enhances the macrophage generation of mitochondrial ROS, as a TLR-generated microbicidal response leading to increased intracellular microbial clearance [104,105]. Silencing as well as chemical inhibition of HDAC by these specific inhibitors induces expression of an orphan nuclear receptor, transcription factor Nur77. It is known to promote anti-inflammatory functions in macrophages by rewiring the tricarboxylic acid (TCA) cycle [106]. Deficiency of Nur77 is associated with increased functional occurrence of proinflammatory phenotype of macrophages, which leads to increased IL-6, 12 and IFN-γ production. Mtb modulates cytokine secretion to invade host defence. Thus, targeting HDACs by counteracting its transcriptional regulation can be quite useful therapeutically [107,108,109]. Along with the epigenetic modulations of HDAC inhibitors, various alternative functions of these agents also contribute to its bactericidal activity [110]. Enhanced anti-Mtb activity of macrophages is dependent on HDAC-6. Its expression is specifically maintained in monocyte-derived macrophages more importantly in patients with resistant Mtb infection. HDAC-6 is also required for maintaining acidic environment in Mtb containing phagosomes in infected macrophages. Thus, it acts as an important novel target for HDT against Mtb [111]. Tubustatin A and HDAC-6 inhibitor (MC2780) significantly inhibit Mtb growth by upregulation of TNFα, IL-12 and IFNγ as well as downregulation of IL-10 [112]. It recruits a greater number of macrophages, DCs and neutrophils, thus inducing delayed innate immune response. RGFP966 is an HDAC3 inhibitor that was also found to modulate pro-inflammatory macrophage secretion by Mtb infected macrophages by decreasing IL-6 and TNF secretion [113]. It induces NOS2, CASP1 as well as the M1 marker CD86. Along with vitamin D3, it leads to two fold induction in cAMP by inhibition of epigenetic mechanism of acetylation during Mtb infection, proving to be an important HDT in TB [97]. SIRT or sirtuins are NAD+ dependent HDACs, which are usually known for their implications on the pathologies of age-related disorders. Owing to the fact that these sirtuins share their targets with HDACs, their inhibitors can be explored for anti-inflammatory properties.The SIRT-1/2 specific inhibitor cambinol was shown to protect against endotoxic and toxic shock via inhibition via the production of pro-inflammatory cytokines in vitro. Thus, SIRT-1/2 might prove to have significant therapeutic potential in Mtb infection [114].

Anacardiac acid is an HAT inhibitor drug which produces isonicotinoylhydrazones on coupling with anti-TB drug isoniazid. These isonicotinoylhydrazones have shown potent inhibitory activity against Mtb [115]. Bromodomains are conserved structural motifs which are associated with chromatin modifying proteins such as HATs [116]. During infection or inflammation, bromodomain inhibitors provide specific targeting along with individual histone post translational modifications. These bromodomain and extra terminal domain family of proteins (BET) link histone acetylation status to the transcription. These drugs are predicted to downregulate systemic inflammation during severe infections by acting at CpG low promotors and allowing modulation of some most potent lipopolysaccharide (LPS) responses, leading to subsequent induction of IL-6, 12 and NO [117]. JQ1-BET (Bromodomain and Extra-Terminal domain), a specific inhibitor of bromodomain, suggests a possible targeted approach with these agents. Another systemic histone, I-BET, reduces the induction of various inflammatory cytokines such as IL-6, 1b, IFNβ following LPS stimulation via TLR4 and TNF-α by activated murine macrophages [118,119].

Glutamate synthase is an enzyme associated with the pathogenicity of Mtb by forming a poly-L glutamate cell wall structure. Thus, phosphorothioate-modified antisense oligodeoxyribonucleotides acting against the mRNA of this enzyme had been shown to reduce the amount of cell wall L-glutamate by 24% and 1.25 log reduction in Mtb growth [120]. A combination of three PS-ODNs, which are 5′-, 3′-hairpin-modified targeting mycolyl transferase transcripts, was each tested in Mtb and shown to reduce the transcription of target gene by 90% and also lead to an 8-fold increase in Mtb sensitivity to isoniazid [121]. Phosphoryl guanidine oligo-2′-O-methylribonucleotides (2′-OMe PGOs) is a novel antisense mRNA targeting molecule that has shown high biological activity by effective intracellular uptake as well as inhibition of target *ald* gene in the infected macrophages [122]. Another thiocationic lipid-based formulation of phosphorothioate antisense oligonucleotides (PAOs) has also shown in vitro efficacy against Mtb [123].

DNA-methylation inhibitor drugs are another class of compounds which target host epigenetics such as 5-azacytidine, which is a cytidine analogue with a nitrogen atom instead of C5 [124]. It is phosphorylated and gets incorporated into the DNA during the process of replication. The process of methylation is widely reduced as a result of irreversible DNMT1-aza linkage, which triggers enzyme degradation [125]. A phase Ib/IIa open label, non-randomized clinical trial (NCT03941496) is being conducted by Andrew DiNardo et al. by using injectable azacytidine in pulmonary TB. A total of 50 participants will be recruited and will be given SQ azacytidine for 25 days in a dose escalation fashion starting from 5 mg/m^2^ up to a maximum of 75 mg/m^2^ 5 days for each dose. Overall incidence and severity of all adverse events as well as measurement of epigenetic mediated immune exhaustion were defined as primary outcomes of the study. The results of this ongoing trial are still in process. Zebularine, another methylation inhibitor drug, shows similar mechanism as azacytidine, where its diphosphate form reactivates the silenced genes after incorporating into the DNA leading to subsequent inhibition of DNA methylation. However, its effectiveness is yet to be proved in TB [126].

CRISPR-associated protein 9 (Cas9), when fused with an acetyltransferase, catalyses the target site acetylation of H3K27, which leads to transcriptional activation of target genes [127]. Apart from this, the CRISPR-Cas9 system, when fused with LSD-1, a histone demethylase, targets various regions of DNA that enhances the expression of a range of genes [128]. This technology has recently been studied, which may prove to be of great importance therapeutically. In this context, Cas9 epigenetic effectors could be used therapeutically as it would be capable to artificially install or remove various epigenetic changes and track the causal effects of the induced epigenetic modifications [129]. Various different drug molecules used in vivo and in vitro are currently being studied to determine whether any modulate the epigenome (Table 3).

## 5. Way Forward

Epigenetic alterations such as histone modifications, alteration in expression of non-coding RNAs, alteration in DNA methylation and miRNA all are interlinked, which determine the disease outcome and pathology, collectively termed as epigenetic processes. The epigenetic changes induced in Mtb are reversible and can be easily rectified with the use of drug modulating epigenetic processes. Some recent successes have been obtained in the use of “epi-drugs” for epigenetic treatment in various cancers [134]. Although epigenetics has a key place in disease outcome and pathology, less research has been conducted as compared to the genomic studies.

The collective knowledge of epigenetic, genomic, proteomic and transcriptomic is required for Mtb-primed immune cells in order to expose the susceptibility of the Mtb pathogen and to target the novel mechanistic pathways and interaction in order to protect the host against the infection. Moreover, the relationships between enzymes such as MMP responsible for cavitation and epigenetic processes such as histone acetylation can be further explored. A recent study considered the effect of a classical anti-Mtb drug isoniazid on epigenetics via post-translational modifications such as iso-nicotinylation at histone sites by increasing the levels of isonicotinyl-CoA. These modifications carried out by isoniazid can lead to malignancies. Thus, through more epigenetic studies, many more mechanisms of such iatrogenic reactions to anti-TB drugs can be explained [135]. The Mtb pathogen also utilizes various epigenetic processes to proliferate in host, which can be targeted effectively as a therapeutic option.

HAT and HDAC are being studied for developing improved regimen and therapeutic alternatives for resistant Mtb cases. In one study, it was noted that over-expression in over 59 miRNAs was responsible for regulating TLR-4 and various pro-inflammatory cytokines. Around 90% of alteration in DNA methylation occurs at cytosine of CpG sites. Moreover, over 23,000 DMRs and also GO analysis had proven the role of non-canonical pathway and the effects of immune cells such as macrophages and DCs in disease induction, respectively, as we know that Mtb and its related products can modulate the epigenetic processes to establish the infection. As mentioned above, the mechanism of PE6 proteins in Mtb can be explored more in order to develop novel HDT strategies for Mtb [136]. Different methods such as pyrosequencing, whole genome bisulfite sequencing, expression of miRNA, chromatin immunoprecipitation and PCR can be studied further and understand of the epigenetic processes in Mtb pathogen. These studies on host epigenetics also explain the immunosuppressive states and their interaction with the Mtb pathogen and how innate and adaptive immunity works, which can help in development of next generation vaccines and various therapeutic regimens. Moreover, the blood analysis for the epigenetically modified products can provide an important tool for the diagnosis of the infection and can serve as a putative biomarker. The mechanisms of hypomethylating agents such as Azacytidine can be targeted on the methylation for Mtb, which leads to specific gene silencing [85]. However, the major issue with targeting epigenetics with agents such as azacytidine is their toxicity. Aza- or sulphur-containing drugs have an established toxicity profile and are known to be intolerable by a large population. Targeting other epigenetic modifications such as histone methylation or acetylation can be safer compared to the use of azacytidine. Thus, a novel epigenetic-targeted therapy ideally should overcome the shortcomings of the currently available treatment regimen for which a lot of other targets as well as agents acting on the existing targets are to be explored.

## 6. Conclusions

Several factors that influence the progression of Mtb infection in the host include environmental factors such as malnutrition, dense population, genetic factors, the host’s immune status and the virulence of the Mtb pathogen. All of these components are interspersed and are challenging to determine causality for each. Hence, the study of the epigenetic processes becomes very important in order to understand the progression of the Mtb. There are various epigenetic processes such as histone modifications, alteration in expression of non-coding RNAs, alteration in DNA methylation and miRNA that can be explored, as they are ideal candidates for a therapeutic target owing to how the host environment induces epigenetic changes. They can also be used to monitor the progression of the disease or the efficacy of the therapy administered. Furthermore, ‘how epigenetics’ influences the activation of the latent Mtb infection and how it can be managed in an infected individuals could be the key to the complete eradication of the Mtb disease.

## Figures and Tables

**Table 1:** Protein and their effects on respective epigenetic changes.

Protein	Epigenetic Target	Effect
Rv3423.1	Histone acetyl transferase	It leads to increase in no. of bacteria in host intracellular environment and their survival [86].
Rv2966c	DNA methylation (non CpG context)	It showed the ability to localize the host’s cell nucleus and repression of specific genes [66].
Rv1988	Histone methyltransferase	It demethylates the amino acid arginine specifically at 42nd position in the histone H3, which has a profound effect on host gene transcription due to its capacity to localize with the chromatin in the host nucleus [54].
Rv3763	Histone acetyl transferase	It is responsible for histone hypoacetylation at CIITA promoter via suppressing IFN-γ induced genes [66].
Rv3263	DNA methylation	It is responsible for all detectable methylation modification by a particular Mtb strain, H37Rv [87].

Abbreviations: CIITA, Class II trans activator; IFN, interferon; Mtb, mycobacterium tuberculosis; Th1, helper T cells.

**Table 2:** Targets affected by epigenetic modifications and therapeutic agents targeting those modifications.

Epigenetic Modifications	Targets Involved	Therapeutic Agents
Histone modifications	MMP IL-12b, IL-1β CIITA, HLA-DR, CD64 NF-κB JAK-STAT pathways	Nonspecific HDAC inhibitors: SAHA (vorinostat) Trichostatin A (TSA) Phenylbutyrate (PBA) HDAC6 specific: Tubustatin A MC2780 HDAC3 specific: RGFP966 Bromodomain inhibitors HAT inhibitor: Anacardiac acid
Alterations in expression of non-coding RNA	DCs TLR4 NF-κB CD1c	Antisense mRNA targeting agents: Phosphorothioate-modified antisense oligodeoxyribonucleotides (PS-ODNs) Phosphoryl guanidine oligo-2′-O-methylribonucleotides (2′-OMe PGOs) Phosphorothioate antisense oligonucleotides (PAOs)
Alteration in DNA methylation	IFN-γ Monocyte derived-DCs TLR2 IL-17, IL-12, IL-10	Methylation inhibitors: 5-Azacytidine Zebularine

Abbreviations: CIITA, Class II trans activator; CD, cluster of differentiation; DC, dendritic cells; HAT, histone acetyltransferases; HDAC, histone deacetylases; HLA, human leukocyte antigen; IFN, interferon; IL, interleukin; JAK-STAT, Janus kinase-signal transducer and activator of transcription; MMP, matrix metalloproteinase; NF-κB, nuclear factor kappa B; SAHA, suberoylandilide hydroxamic acid; TLR, toll-like receptors.

**Table 3:** Summary of drugs affecting epigenetics in various in vivo and in vitro models of tuberculosis.

Target of the Therapy	Therapeutic Agent	Nature of STUDY	Results
HDAC non-specific inhibitors	Vorinostat Valproic acid	In vitro (H37Rv cultures)	Vorinostat and Valproic acid showed 1.5 and 2 log reduction in CFU, respectively. Both improved the efficacy of rifampicin against Mtb [130].
	Trichostatin A	In vivo (*M. marinum* infected zebrafish)	32% reduction of bacterial burden in pre-treated model [98].
	Phenylbutyrate with Vitamin D	In vivo (Humans)	Decline in concentrations of cytokines such as TNFα, CCL11/5, PDGF-β Increase in LC3 expression [131]
	Phenylbutyrate with Vitamin D	In vivo (Humans- RCT)	28.8% increase in rate of patients’ culture conversion compared to placebo [132].
HDAC 6 inhibitors	Tubastatin A	In vivo (C57BL/6 mice model infected with Mtb)	6 log reduction in CFU after 14 days Upregulation of TNFα, IL-12, IFNγ and downregulation of IL-10 [112].
	Tubastatin A	In vitro (THP-1 cell line)	Inhibition of TNFα and IL-6 in the cell line [105].
Bromodomain inhibitor	CBP30	In vitro (heparinized human blood)	No effect on expression of TNFα [133].
HAT inhibitors	Isonicotinoylhydrazones derived from anacardic acid	In vitro (*M. smegmatis* mc2155 cells)	MIC of 4 µg/mL Synergistic actions with isoniazid [115].
DNA methylation inhibitors	Azacytidine	In vivo (Humans)	The trial is still ongoing (NCT03941496)

Abbreviations: CCL-11; C-C motif chemokine-11, CFU; colony forming units, DNA; Deoxyribose nucleic acid, HAT; Histone acetyl transferase, HDAC; Histone deacetylase, IFN-γ; interferon-γ, IL-12; interleukin-12, LC-3; microtubule associated protein light chain-3, MIC; minimum inhibitory concentration, Mtb; mycobacterium tuberculosis, PDGF-β; platelet derived growth factor-β, RCT; randomized control trial, TNF-α; tumour necrosis factor-α.

## Data Availability

Not applicable.
